# An Unusual Presentation of Isolated Contrecoup Injury

**DOI:** 10.7759/cureus.89141

**Published:** 2025-07-31

**Authors:** Thomas Francis, Dhritiman Chakrabarti, Sarina K, Ivy George

**Affiliations:** 1 Department of Neuroanesthesiology and Neurocritical Care, National Institute of Mental Health and Neurosciences, Bengaluru, IND

**Keywords:** contrecoup injury, mesh cranioplasty, neurosurgery, subdural hematoma, traumatic brain injury

## Abstract

A 38-year-old man sustained a traumatic brain injury (TBI) following a road traffic accident, presenting unconscious with vomiting and right ear bleeding. He had a prior history of head trauma with cranioplasty. On admission, he was deeply unconscious (Glasgow Coma Scale (GCS) E1VTM3) with unequal non-reactive pupils. Imaging revealed a significant left-sided acute subdural hematoma (SDH), burst temporal lobe, and midline shift, alongside an old cranioplasty mesh and right petrous bone fracture. Notably, there was minimal injury on the right (impact) side but extensive hemorrhagic damage on the left (opposite) side, suggesting a contrecoup injury. He underwent emergency decompressive craniotomy and postoperative ICU care, eventually recovering enough for discharge.

This case highlights an unusual presentation of contrecoup injury in the absence of significant coup-related hemorrhage. The presence of a cranioplasty mesh likely influenced injury mechanics. The proposed mechanism involves both positive and negative pressure theories: the brain lagging behind skull motion (positive pressure) and rebound forces (negative pressure), creating damage on the opposite side. The mesh’s rigidity compared to natural bone may have amplified injury through stress concentration at the mesh-bone interface. This unique biomechanical interaction may explain the disproportionate injury seen contralaterally.

To our knowledge, no previous case reports describe such an isolated contrecoup injury pattern associated with a cranioplasty mesh. This report underscores the importance of considering altered skull biomechanics in patients with prior cranial surgeries and contributes novel insight into TBI dynamics.

## Introduction

Traumatic brain injury (TBI) remains a major global health burden, significantly contributing to both mortality and long-term disability. Among patients with severe TBI, focal brain injuries are identified in nearly 50% of cases and are responsible for approximately two-thirds of TBI-related deaths. One specific type of focal injury, the contrecoup injury, occurs at a site anatomically opposite to the primary point of impact.

The pathophysiology of contrecoup injuries has been partially explained by the shock wave theory, which suggests that mechanical forces generated at the site of impact propagate through intracranial structures, resulting in stress concentration or cavitation at distant sites. This mechanism often leads to more widespread parenchymal damage compared to coup injuries alone. Clinically, contrecoup injuries are associated with increased severity at presentation, frequently evidenced by a lower Glasgow Coma Scale (GCS), and are linked to poorer prognostic outcomes. Epidemiological data indicate that contrecoup injuries occur in approximately 10% of TBI cases, with a mean affected age of 38.5 years. Road traffic accidents constitute the most common mechanism of injury, with acute subdural hematoma (SDH) being the predominant lesion, followed by hemorrhagic contusions. Reported mortality rates in this population range from 43% to 53% [1,2].

Cranioplasty, used to repair skull defects, can involve autologous bone or synthetic mesh. Autologous cranioplasty uses the patient’s own bone, offering good biocompatibility and lower cost, but carries risks like bone resorption and infection. Mesh cranioplasty uses materials like titanium, which are readily available and resistant to resorption, though they are more expensive and may carry a risk of foreign body reaction. The choice depends on patient factors and clinical context.

The contrecoup injuries are of particular interest in the setting of post-cranioplasty trauma, where the presence of implanted materials may modify the distribution and absorption of mechanical forces, potentially influencing injury patterns.

## Case presentation

A 38-year-old man was riding a two-wheeler vehicle when he was involved in an unwitnessed road traffic accident and was found unconscious on the roadside. He had multiple episodes of vomiting and bleeding from the right ear. He also had an abrasion on the right lateral malleolus. The patient had a history of a head injury 10 years ago. On admission to the hospital, his GCS was E1VTM3 with unequal non-reactive pupils (2 mm on the right side and 3 mm on the left side) with abnormal flexion of the limbs. He was not on any anticoagulants. He had no known comorbidities.

**Figure 1:**
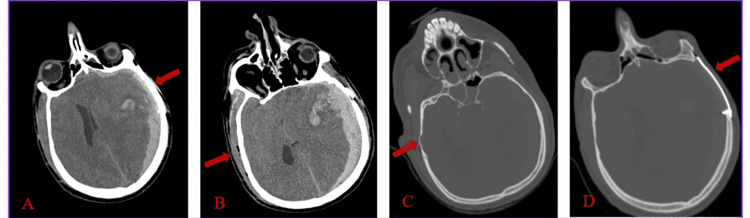
CT brain findings A: left thick acute subdural hematoma with contusion and midline shift (red arrow); B: the air pockets in the subcutaneous tissue of the right side (red arrow); C: bone window shows fracture of the petrous bone on the right side (red arrow); D: bone window shows left side mesh (red arrow)

Cranial computed tomography (CT) showed a left frontotemporoparietal thick acute SDH with temporal lobe involvement and a midline shift of 1.5 cm to the right side. Also, an old cranioplasty mesh was seen on the left frontotemporoparietal side with left frontal gliosis. Bone-window CT with 5 mm slice thickness demonstrated a visible fracture in the right petrous bone with overlying air pockets seen in the subcutaneous tissue (Figure 1).

He underwent extension of the left frontal craniotomy and left frontotemporoparietal decompressive craniotomy, evacuation of acute SDH, and augmentative duroplasty with a bone flap inserted in the abdomen. Intraoperatively, it was observed that the mesh was made of titanium. After surgery, he was shifted to the ICU for sedation and ventilation. The patient was gradually weaned from the ventilator and extubated, and discharged with a GCS of E4V4M5.

## Discussion

Fractures of the petrous part of the temporal bone are typically associated with coup injuries. However, in this case, there were no significant hemorrhagic findings on the right (impact) side, whereas a substantial acute SDH with contusion was observed on the left side. This pronounced contralateral injury may be explained by a unique mechanism involving the presence of a cranioplasty mesh on the left side. We propose that the disproportionate severity of the contrecoup injury, in the absence of notable coup damage, can be understood through a combination of the positive and negative pressure theories [3].

According to the positive pressure theory, during sudden deceleration, the brain lags behind the skull, resulting in compression of the brain against the contralateral internal surface of the skull and, in this case, the rigid mesh. Conversely, the negative pressure theory suggests that the brain's movement in one direction generates tensile stress on the opposite side, leading to tissue disruption. The extent of damage observed in this patient may have been amplified by the differences in mechanical properties (elastance) between the native skull and the cranioplasty mesh. Another potential contributing factor to the injury is the involvement of the screw. Imaging suggests that the injury extends into the brain parenchyma, indicating that the sharp structure of the screw may have damaged adjacent blood vessels upon impact, thereby exacerbating the injury in conjunction with the mesh. The combined presence of the mesh and penetrating screws may have played a significant role in the development of the extensive contrecoup injury observed in this case. To the best of our knowledge, to date, no published case reports have specifically described injuries resulting from polymethylmethacrylate (PMMA), titanium, or autologous cranioplasty. Based on our findings, the extent of injury appears to be greater with the use of titanium mesh. However, additional case reports are necessary to enable a comparative analysis of the injuries associated with different cranioplasty materials. To date, 22 cases of contrecoup acute EDH without fracture have been reported [4-6]. To the best of our knowledge, no prior case report has documented such a mechanism of injury.

## Conclusions

We report a rare case of contrecoup brain injury characterized by the interaction between the native skull and a cranioplasty mesh, resulting in minimal intracerebral injury at the site of impact and pronounced damage at the contralateral site. We also discuss the potential underlying mechanisms contributing to this injury pattern. The ideal care for contrecoup brain injury post-cranioplasty includes rapid stabilization, urgent imaging, and decompressive craniotomy. Post-op ICU care focuses on sedation, intracranial pressure (ICP) control, seizure prevention, and neuroprotection. Cranioplasty materials like titanium mesh may worsen injury, so long-term management should include rehab and careful re-cranioplasty planning.

## References

[1] Jayakumar PN, Sastry Kolluri VR, Basavakumar DG, Subbakrishna DK, Arya BY, Das BS, Narayana Reddy GN (1991). Prognosis in contre-coup intracranial haematomas--a clinical and radiological study of 63 patients. Acta Neurochir (Wien).

[2] Banga MS, Sandeep BV, Roy K, Saha SK, Dixit S, Ghosh P (2017). Contrecoup head injury. Indian J Neurosurg.

[3] Drew LB, Drew WE (2004). The contrecoup-coup phenomenon. Neurocrit Care.

[4] Musali SR, Manne S, Butkuri N, Gollapudi PR, Kumar TS (2019). Contrecoup extradural hematoma without fracture: a case report and review of literature. Asian J Neurosurg.

[5] Bal'afif F, Wardhana DW, Alfandy TN, Jesse A (2022). Contrecoup epidural hematoma: a rare case report. Pan Afr Med J.

[6] Andoh S, Matsuura C, Sakaeyama Y (2018). Acute contrecoup epidural hematoma that developed without skull fracture in two adults: two case reports. J Med Case Rep.

